# Population Health Metrics During the Early Stages of the COVID-19 Pandemic: Correlative Pilot Study

**DOI:** 10.2196/40215

**Published:** 2022-10-17

**Authors:** Marie A Severson, David A Cassada, Victor C Huber, Daniel D Snow, Lisa M McFadden

**Affiliations:** 1 Division of Basic Biomedical Sciences University of South Dakota Vermillion, SD United States; 2 Water Sciences Laboratory University of Nebraska–Lincoln Lincoln, NE United States

**Keywords:** COVID-19, ethyl glucuronide, wastewater, stress, helpline

## Abstract

**Background:**

COVID-19 has caused nearly 1 million deaths in the United States, not to mention job losses, business and school closures, stay-at-home orders, and mask mandates. Many people have suffered increased anxiety and depression since the pandemic began. Not only have mental health symptoms become more prevalent, but alcohol consumption has also increased during this time. Helplines offer important insight into both physical and mental wellness of a population by offering immediate, anonymous, cheap, and accessible resources for health and substance use disorders (SUD) that was unobstructed by many of the mandates of the pandemic. Further, the pandemic also launched the use of wastewater surveillance, which has the potential for tracking not only population infections but also consumption of substances such as alcohol.

**Objective:**

This study assessed the feasibility of using multiple public surveillance metrics, such as helpline calls, COVID-19 cases, and alcohol metabolites in wastewater, to better understand the need for interventions or public health programs in the time of a public health emergency.

**Methods:**

Ethanol metabolites were analyzed from wastewater collected twice weekly from September 29 to December 4, 2020, in a Midwestern state. Calls made to the helpline regarding housing, health care, and mental health/SUD were correlated with ethanol metabolites analyzed from wastewater samples, as well as the number of COVID-19 cases during the sampling period.

**Results:**

Correlations were observed between COVID-19 cases and helpline calls regarding housing and health care needs. No correlation was observed between the number of COVID-19 cases and mental health/SUD calls. COVID-19 cases on Tuesdays were correlated with the alcohol metabolite ethyl glucuronide (EtG). Finally, EtG levels were negatively associated with mental health/SUD helpline calls.

**Conclusions:**

Although helpline calls provided critical services for health care and housing-related concerns early in the pandemic, evidence suggests helpline calls for mental health/SUD-related concerns were unrelated to COVID-19 metrics. Instead, COVID metrics were associated with alcohol metabolites in wastewater. Although this research was formative, with continued and expanded monitoring of population metrics, such as helpline usage, COVID-19 metrics, and wastewater, strategies can be implemented to create precision programs to address the needs of the population.

## Introduction

The SARS-CoV-2 pandemic has caused tremendous stress in the United States. Widespread unemployment and financial strain [1,2], orders to shelter in place [3], health care infrastructure stress [4], and disruptions to education caused significant distress and uncertainty for many people in the United States. National surveys have found that anxiety and depression increased during the pandemic [5]. Early in the pandemic, serious psychological distress was 3.5 times higher in April 2020 compared to 2018 in a nationally representative survey of adults [6]. Similarly, a national survey by the US Census Bureau found that anxiety and depression were 4 times higher in 2020 compared to 2019 [7]. These levels of anxiety and depressive symptoms persisted into the fall of 2020, when COVID-19 cases began to increase. Overall, the negative impacts of COVID-19 on mental health have been well documented [8-11].

Although psychological distress persisted, people changed their activities. Emergency department employees reported decreased exercise and increased alcohol consumption [4]. Increased alcohol consumption was reported in other studies as well. A 51% increase in alcohol sales occurred during the early weeks of the pandemic [12]. Across the United States, beer, wine, and liquor store sales increased by 21.5% in October 2020 compared to the previous 3 years. Elevated alcohol sales continued into November and December 2020 when compared to the average sales of the 3 prior years [13]. Similarly, alcohol consumption increased in 2 independent surveys [8,10]. This increase was greater in females compared to males [12,14]. Urban college students also reported more alcohol consumption following the start of the pandemic [15]. The shift in mental health and the rise in alcohol use since the beginning of the pandemic may highlight the critical need for connecting people with resources for mental health concerns. However, given the stress of the pandemic on the health care infrastructure and subsequent restrictions in face-to-face appointments, population metrics of alcohol use that are less influenced by pandemic-associated restrictions are critically needed. Wastewater analysis of alcohol metabolites provides a community-wide assessment of alcohol use, which does not depend upon restrictions imposed by the pandemic.

Helplines are a critical source of information during times of crisis. These helplines provide immediate, anonymous, cheap, and accessible resources for mental health and substance use disorders (SUD) [16]. Moreover, during the SARS-CoV-2 pandemic, helplines overcame many of the restrictions imposed with traditional face-to-face services, thus allowing them to function unobstructed during these times. Within the United States, few studies have examined the use of helplines during a national emergency, and most have focused on suicidality or depression [17,18]. One study by Brülhart et al [16] examined helpline calls during the SARS-CoV-2 pandemic in multiple countries, including the United States. Findings revealed a significant increase in call volume 6 weeks after the first major outbreak [16]. Subsequent analysis from France and Germany found increased call volumes related to infections and loneliness but only a small decrease in call volumes related to SUD. Subsequent waves revealed no significant change in call volumes related to SUD. However, understanding helpline use during the pandemic in the United States remains underinvestigated.

The purpose of this study was to examine the relationships among population health metrics during a public health emergency. Specifically, we compared COVID-19 cases with calls to a regional helpline, including inquiries related to housing, health care, and mental health/SUD needs. We also compared these variables with alcohol metabolites found in wastewater samples during this time to estimate population alcohol use. Findings provide the feasibility of these public health–monitoring systems to improve insights into the specific needs for programs to address population health during a public health emergency.

## Methods

### Ethical Considerations

To reduce the risk of identifying communities, metrics as suggested by Prichard et al [19] were used. Only the broad geographical location (state) was given. These efforts aligned with ethical guidelines for wastewater-based epidemiology set forth by the Sewage Analysis Core group Europe [20] to help minimize risk to participating communities and their citizens. Similarly, given that county-level metrics would provide identifiable cities, only correlations among metrics were included. Helpline calls and COVID-19 case metrics were considered exempt from human subjects’ ethical review under the federal regulations for human subjects (45 CFR Part 46) because (1) the data came from sources that are publicly available and (2) the data were deidentified and uncoded and stripped of identifiers [21].

### Wastewater Samples

Twenty-four-hour composite wastewater samples were collected at the inlet of a wastewater treatment plant at approximately 9:00 a.m. on Tuesdays and Fridays, September 29-December 4, 2020. Approximately 500 mL of wastewater was frozen until analysis. Samples were shipped to the Water Sciences Laboratory at the University of Nebraska-Lincoln for analysis of ethyl glucuronide (EtG) and ethyl sulfate (EtS) by multiple reaction monitoring using ultrahigh-pressure liquid chromatography coupled with tandem quadrupole mass spectrometry (UHPLC-MS-MS).

### Analysis of Ethanol Metabolites

Sulfate (EtS) and glucuronide (EtG) metabolites of ethanol were analyzed by isotope dilution using a Xevo TQ-S micro triple quadrupole mass analyzer interfaced with an Acquity UHPLC and UniSprayTM ionization source in a negative-ion-detection-mode spectrometer (Waters Corporation, Milford, MA). An Acquity BEH C18 column (2.1 mm × 50 mm, 1.7 µm) was used for separation at a flow rate of 0.6 mL/minute and a temperature of 40 °C. In addition, 0.1% (v/v) formic acid with 1.0 g/L of ammonium formate in water (solvent A) and a 50/50 (v/v) methanol/acetonitrile (solvent B) binary gradient was used, as listed in Table 1. The injection volume was 2.0 µL. Mass spectrometer settings included the following: desolvation gas temperature=600 °C, desolvation gas flow=1000 L/hour (N_2_), cone gas flow=50 L/hour (N_2_), impactor voltage=1.0 kV, LM1 resolution=9.2, HM1 resolution=15.1, ion energy 1=0.1 V, LM2 resolution=9.0, HM2 resolution=15.1, and ion energy 2=0.6 V. Retention times and mass scan segments are listed in Table 2. The UHPLC-MS-MS equipment was controlled by MassLynx software (version 4.2 SCN 1017, Waters Corporation). Aqueous standards were prepared by dilution of analytical standards for deuterium-labeled and nonlabeled EtG and EtS (certified reference material, Cerilliant, Sigma-Aldrich, St Louis, MO, USA) in purified organic-free reagent water over the range of 1.0-200 ng/mL. Instrument detection limits, estimated as 3 times the SD of the lowest calibration standard, were 0.93 and 0.23 for EtG and EtS, respectively. Syringe-filtered (0.2 µm pore size) samples were spiked with internal standards and analyzed without additional preparation.

**Table 1:** Gradient setting for UHPLC-MS-MS^a^ analysis at a flow rate of 0.6 mL/minute. All solvent transitions were linear.

Time (minutes)	0.3% (v/v) formic acid/1.0 g/L of ammonium formate/H2O	50/50 (v/v) methanol/acetonitrile
0	100%	0%
1.0	100%	0%
2.0	50%	50%
2.5	0%	100%
5.75	0%	100%
6.0	100%	0%
9.0	100%	0%

^a^UHPLC-MS-MS: ultrahigh-pressure liquid chromatography coupled with tandem quadrupole mass spectrometry.

**Table 2:** Retention times and mass scan settings.

Compound	Retention time (minutes)	Precursor ion (amu)	Product ion (amu)	Dwell time (ms)	Fragmentation voltage (V)	Collision energy (V)
EtS^a^	0.34	124.9	96.9	23	40	11
d5-EtS	0.34	129.9	97.9	23	40	11
EtG^b^	0.55	220.9	84.9	23	40	15
d5-EtG	0.54	225.9	84.9	23	40	15

^a^EtS: ethyl sulfate.

^b^EtG: ethyl glucuronide.

### Helpline Call Statistics

The South Dakota Helpline Center is a free resource for those in need of assistance. People may call, text, or email the helpline center about their needs 24 hours a day, 7 days a week. Each contact is classified by the needs expressed during the call, including but not limited to needs for housing, health care, food/meals, and mental health/SUD assistance. Data on daily calls and call categories are posted on the interactive website [22]. To ensure caller privacy, categories of calls below a minimum threshold are suppressed. To ensure that data exceeded the threshold to be categorized, calls were aggregated to align with wastewater sampling (sum of calls from Saturday to Tuesday, sum of calls from Wednesday to Friday). Calls during the sampling period were included in the analysis described.

### COVID-19 Cases

The South Dakota Department of Health reported positive COVID-19 clinical cases daily during the sampling period. Positive cases included only South Dakota residents regardless of where they were tested. Cases were reported by the county of residence. Case counts and 7-day averages were reported daily [23].

### Alcohol Metabolites

Sulfate (EtS) and glucuronide (EtG) metabolites of ethanol were used as a population estimate of alcohol use. Wastewater epidemiology is a well-established metric of human drug consumption [24]. Chen et al [25] have previously shown that wastewater estimates of alcohol consumption were in good agreement with US survey data based on sampling from 3 communities over a 1-year period.

### Statistics

SAS Studio was used for bivariate Pearson 2-tailed correlational analysis. Violations of assumptions were not observed; therefore, parametric analysis was used. Variables (helpline: total calls, health care, housing, and mental health/SUD related; COVID-19: county cases, county 7-day average cases, and state 7-day average cases; alcohol metabolites: EtS and EtG) were first correlated across all dates (N=19) and then by day of the week (Tuesday: n=10, 53%; Friday: n=9, 47%). Significance was set at *P*<.05.

## Results

### Correlations Among COVID-19 Cases, Alcohol Metabolites, and Helpline Calls

When all sampling dates were included (Table 3), calls to the helpline were significantly positively correlated with measures of COVID-19 cases, including new county cases (*t*_17_=2.90, *P*=.01), county 7-day moving average (*t*_17_=6.87, *P*<.001), and state 7-day moving average (*t*_17_=9.83, *P*<.001). More specifically, measures of COVID-19 cases were significantly correlated with helpline calls related to health care (county 7-day average: *t*_17_=3.13, *P*=.01; state 7-day average: *t*_17_=3.07, *P*=.01) and housing (county 7-day average: *t*_17_=3.28, *P*=.004; state 7-day average: *t*_17_=3.56, *P*=.002) but not mental health/SUD (county 7-day average: *t*_17_=–1.08, *P*=.29; state 7-day average: *t*_17_=–1.06, *P*=.30). In contrast, helpline calls related to mental health/SUD were significantly negatively correlated with wastewater EtG (*t*_17_=2.29, *P*=.03) but not EtS (*t*_17_=0.10, *P*=.92) levels.

When examining measures by day of the week, measures of COVID-19 cases were still significantly correlated with total helpline calls on Tuesdays (county cases: *t*_8_=2.70, *P*=.03; county 7-day average: *t*_8_=4.70, *P*=.002; state 7-day average: *t*_8_=6.73, *P*<.001; Table 4) and Fridays (county 7-day average: *t*_7_=4.43, *P*=.003; state 7-day average: *t*_7_=7.10, *P*<.001; Table 5) except for new county cases (Friday: *t*_7_=1.06, *P*=.33; Table 5). On Fridays, county COVID cases (*t*_7_=3.06, *P*=.02), county moving average (*t*_7_=5.47, *P*<.001), and state moving average (*t*_7_=3.72, *P*=.01) were significantly positively correlated with calls related to health care but not housing (county cases: *t*_7_=0.22, *P*=.83; county 7-day average: *t*_7_=1.47, *P*=.18; state 7-day average: *t*_7_=2.09, *P*=.08) or mental health/SUD (county cases: *t*_7_=0.43, *P*=.68; county 7-day average: *t*_7_=0.99, *P*=.36; state 7-day average: *t*_7_=1.36, *P*=.21). In contrast, on Tuesdays, county and state moving average COVID-19 cases were significantly correlated with helpline calls for housing (county 7-day average: *t*_8_=2.85, *P*=.02; state 7-day average: *t*_8_=2.71, *P*=.03) but not health care (county 7-day average: *t*_8_=1.58, *P*=.15; state 7-day average: *t*_8_=1.79, *P*=.11) or mental health/SUD (county 7-day average: *t*_8_=0.57, *P*=.58; state 7-day average: *t*_8_=0.32, *P*=.76). Further, county cases were significantly positively correlated with EtG (*t*_8_=2.63, *P*=.03). EtG was negatively correlated with helpline calls related to mental health/SUD (*t*_8_=2.82, *P*=.02) on Tuesdays. Similarly, on Fridays, EtG was negatively correlated with helpline calls related to mental health/SUD but was only marginally associated (*t*_7_=–2.13, *P*=.07).

**Table 3:** Correlations among COVID-19 cases, alcohol metabolites, and helpline calls (N=19).

Measures		County cases	County 7-day average	SD 7-day average	EtG^a^	EtS^b^	SD helpline total calls	SD helpline-health care	SD helpline-housing	SD helpline- mental health/SUD^c^
**County cases**										
	r	1.00	0.78^d^	0.68^d^	0.03	–0.07	0.58^d^	0.45	0.34	–0.13
	*t*_17_	N/A^e^	5.07	3.86	0.13	–0.30	2.90	2.07	1.49	–0.53
	*P* value	N/A	<.001	.001	.90	.77	.01	.05	.16	.60
**County 7-day average**										
	r	0.78^d^	1.00	0.93^d^	0.24	–0.09	0.86^d^	0.61^d^	0.62^d^	–0.25
	*t*_17_	5.07	N/A	10.38	1.00	–0.35	6.87	3.13	3.28	–1.08
	*P* value	<.001	N/A	<.001	.33	.73	<.001	.01	.004	.29
**SD 7-day average**										
	r	0.68^d^	0.93^d^	1.00	0.28	–0.06	0.92^d^	0.60^d^	0.65^d^	–0.25
	*t*_17_	3.86	10.38	N/A	1.20	–0.26	9.83	3.07	3.56	–1.06
	*P* value	.001	<.001	N/A	.25	.80	<.001	.01	.002	.30
**EtG**										
	r	0.03	0.24	0.28	1.00	0.34	0.19	–0.01	0.08	–0.49^d^
	*t*_17_	0.13	1.00	1.20	N/A	1.51	0.78	–0.05	0.31	–2.29
	*P* value	.90	.33	.25	N/A	.15	.44	.96	.76	.03
**EtS**										
	r	–0.07	–0.09	–0.06	0.34	1.00	–0.05	–0.11	–0.12	0.02
	*t*_17_	–0.30	–0.35	–0.26	1.51	N/A	–0.19	–0.46	–0.49	0.10
	*P* value	.77	.73	.80	.15	N/A	.85	.65	.63	.92
**SD helpline total calls**										
	r	0.58^d^	0.86^d^	0.92^d^	0.19	–0.05	1.00	0.72^d^	0.67^d^	–0.21
	*t*_17_	2.90	6.87	9.83	0.78	–0.19	N/A	4.24	3.68	–0.89
	*P* value	.01	<.001	<.001	.44	.85	N/A	<.001	.002	.39
**SD helpline-health care**										
	r	0.45	0.61^d^	0.60^d^	–0.01	–0.11	0.72^d^	1.00	0.16	–0.34
	*t*_17_	2.07	3.13	3.07	–0.05	–0.46	4.24	N/A	0.66	–1.50
	*P* value	.05	.01	.01	.96	.65	<.001	N/A	.52	.15
**SD helpline-housing**										
	r	0.34	0.62^d^	0.65^d^	0.08	–0.12	0.67^d^	0.16	1.00	0.05
	*t*_17_	1.49	3.28	3.56	0.31	–0.49	3.68	0.66	N/A	0.20
	*P* value	.16	.004	.002	.76	.63	.002	.52	N/A	.84
**SD helpline-mental health/SUD**										
	r	–0.13	–0.25	–0.25	–0.49^d^	0.02	–0.21	–0.34	0.05	1.00
	*t*_17_	–0.53	–1.08	–1.06	–2.29	0.10	–0.89	–1.50	0.20	N/A
	*P* value	.60	.29	.30	.03	.92	.39	.15	.84	N/A

^a^EtG: ethyl glucuronide.

^b^EtS: ethyl sulfate.

^c^SUD: substance use disorders.

^d^*P*<.05

^e^N/A: not applicable.

**Table 4:** Tuesday correlations among COVID-19 cases, alcohol metabolites, and helpline calls (n=10).

Measures		County cases			County 7-day average		SD 7-day average		EtG^a^		EtS^b^		SD helpline total calls	SD helpline-health care			SD helpline-housing		SD helpline- mental health/SUD^c^
**County cases**																			
	r		1.00	0.89^d^		0.84^d^		0.68^d^		0.04		0.69^d^			0.32	0.54		–0.27	
	*t*_8_		N/A^e^	5.39		4.30		2.63		0.11		2.70			0.97	1.83		–0.80	
	*P* value		N/A	<.001		.003		.03		.92		.03			.36	.11		.44	
**County 7-day average**																			
	r		0.89^d^	1.00		0.91^d^		0.60		0.03		0.86^d^			0.49	0.71^d^		–0.20	
	*t*_8_		5.39	N/A		6.02		2.10		0.09		4.70			1.58	2.85		–0.57	
	*P* value		<.001	N/A		<.001		.07		.93		.002			.15	.02		.58	
**SD 7-day average**																			
	r		0.84^d^	0.91^d^		1.00		0.42		–0.04		0.92^d^			0.54	0.69^d^		–0.11	
	*t*_8_		4.30	6.02		N/A		1.30		–0.11		6.73			1.79	2.71		–0.32	
	*P* value		.003	<.001		N/A		.23		.92		<.001			.11	.03		.76	
**EtG**																			
	r		0.68^d^	0.60		0.42		1.00		–0.03		0.32			0.26	0.12		–0.71^d^	
	*t*_8_		2.63	2.10		1.30		N/A		–0.07		0.95			0.75	0.34		–2.82	
	*P* value		.03	.07		.23		N/A		.94		.37			.48	.75		.02	
**EtS**																			
	r		0.04	0.03		–0.04		–0.03		1.00		0.06			0.22	–0.29		–0.39	
	*t*_8_		0.11	0.09		–0.11		–0.07		N/A		0.17			0.63	–0.86		–1.20	
	*P* value		.92	.93		.92		.94		N/A		.87			.55	.41		.27	
**SD helpline total calls**																			
	r		0.69^d^	0.86^d^		0.92^d^		0.32		0.06		1.00			0.74^d^	0.60		–0.14	
	*t*_8_		2.70	4.70		6.73		0.95		0.17		N/A			3.11	2.13		–0.39	
	*P* value		.03	.002		<.001		.37		.87		N/A			.01	.07		.71	
**SD helpline-health care**																			
	r		0.32	0.49		0.54		0.26		0.22		0.74^d^			1.00	0.14		0.46	
	*t*_8_		0.97	1.58		1.79		0.75		0.63		3.11			N/A	0.40		–1.45	
	*P* value		.36	.15		.11		.48		.55		.01			N/A	.70		.18	
**SD helpline-housing**																			
	r		0.54	0.71^d^		0.69^d^		0.12		0.29		0.60			0.14	1.00		0.21	
	*t*_8_		1.83	2.85		2.71		0.34		–0.86		2.13			0.40	N/A		0.62	
	*P* value		.11	.02		.03		.75		.41		.07			.70	N/A		.55	
**SD helpline-mental health/SUD**																			
	r		0.27	0.20		0.11		0.71^d^		0.39		0.14			0.46	0.21		1.00	
	*t*_8_		–0.80	–0.57		–0.32		–2.82		–1.20		–0.39			–1.45	0.62		N/A	
	*P* value		.44	.58		.76		.02		.27		.71			.18	.55		N/A	

^a^EtG: ethyl glucuronide.

^b^EtS: ethyl sulfate.

^c^SUD: substance use disorders.

^d^*P*<.05

^e^N/A: not applicable.

**Table 5:** Friday correlations among COVID-19 cases, alcohol metabolites and helpline calls (n=9).

Measures		County cases	County 7-day average	SD 7-day average	EtG^a^	EtS^b^	SD helpline total calls	SD helpline-health care	SD helpline-housing	SD helpline- mental health/SUD^c^	
**County cases**											
	r	1.00	0.60	0.48	0.39	0.02	0.37	0.76^d^	0.08	0.16	
	*t*_7_	N/A^e^	1.99	1.46	–1.11	–0.04	1.06	3.06	–0.22	0.43	
	*P* value	N/A	.09	.19	.30	.97	.33	.02	.83	.68	
**County 7-day average**											
	r	0.60	1.00	0.97^d^	0.18	0.15	0.86^d^	0.90^d^	0.49	0.35	
	*t*_7_	1.99	N/A	10.02	0.50	–0.40	4.43	5.47	1.47	–0.99	
	*P* value	.09	N/A	<.001	.63	.70	.003	<.001	.18	.36	
**SD 7-day average**											
	r	0.48	0.97^d^	1.00	0.31	0.19	0.94^d^	0.82^d^	0.62	0.46	
	*t*_7_	1.46	10.02	N/A	0.87	–0.50	7.10	3.72	2.09	–1.36	
	*P* value	.19	<.001	N/A	.41	.63	<.001	.01	.08	.21	
**EtG**											
	r	0.39	0.18	0.31	1.00	0.37	0.32	0.06	0.22	0.63	
	*t*_7_	–1.11	0.50	0.87	N/A	–1.06	0.90	0.15	0.59	–2.13	
	*P* value	.30	.63	.41	N/A	.32	.40	.88	.58	.07	
**EtS**											
	r	0.02	0.15	0.19	0.37	1.00	0.01	0.06	0.03	0.33	
	*t*_7_	–0.04	–0.40	–0.50	–1.06	N/A	–0.04	–0.15	–0.09	0.94	
	*P* value	.97	.70	.63	.32	N/A	.97	.88	.93	.38	
**SD helpline total calls**											
	r	0.37	0.86^d^	0.94^d^	0.32	0.01	1.00	0.71^d^	0.77^d^	0.34	
	*t*_7_	1.06	4.43	7.10	0.90	–0.04	N/A	2.67	3.22	–0.97	
	*P* value	.33	.003	<.001	.40	.97	N/A	.03	.01	.36	
**SD helpline-health care**											
	r	0.76^d^	0.90^d^	0.82^d^	0.06	0.06	0.71^d^	1.00	0.17	0.10	
	*t*_7_	3.06	5.47	3.72	0.15	–0.15	2.67	N/A	0.47	–0.25	
	*P* value	.02	<.001	.01	.88	.88	.03	N/A	.65	.81	
**SD helpline-housing**											
	r	0.08	0.49	0.62	0.22	0.03	0.77^d^	0.17	1.00	0.27	
	*t*_7_	–0.22	1.47	2.09	0.59	–0.09	3.22	0.47	N/A	–0.75	
	*P* value	.83	.18	.08	.58	.93	.01	.65	N/A	.48	
**SD helpline-mental health/SUD**											
	r	0.16	0.35	0.46	0.63	0.33	0.34	0.10	0.27	1.00	
	*t*_7_	0.43	–0.99	–1.36	–2.13	0.94	–0.97	–0.25	–0.75	N/A	
	*P* value	.68	.36	.21	.07	.38	.36	.81	.48	N/A	

^a^EtG: ethyl glucuronide.

^b^EtS: ethyl sulfate.

^c^SUD: substance use disorders.

^d^*P*<.05

^e^N/A: not applicable.

## Discussion

### Principal Findings

This study assessed the feasibility of using multiple public surveillance metrics, such as helpline calls, COVID-19 cases, and wastewater alcohol metabolites, to better understand public health and the need for interventions at the time of a public health emergency. Evidence supports that the helpline provided critical support for people navigating the pandemic. Multiple metrics of clinical COVID-19 cases were positively correlated with total helpline calls and helpline calls regarding health care. Moreover, the helpline was an essential resource to assist people experiencing housing insecurities that may have been associated with economic downturns related to the pandemic. However, COVID-19 cases were not significantly correlated with helpline calls for mental health and SUD resources. Instead, county COVID-19 cases were significantly associated with the alcohol metabolite EtG. Further, on Tuesdays, helpline calls regarding mental health/SUD were negatively correlated with EtG levels. We speculate that these findings suggest that people may have been using alcohol to cope with the stressors associated with the pandemic, especially during the week.

Nationwide, studies have suggested increases in fear, anxiety, and depression since the beginning of the pandemic. According to the US Census Household Pulse Survey 2020-2022, a national average of 40.14% of participants reported symptoms of either anxiety or depression during a similar sampling period [5]. Nationally reported symptoms peaked on November 11-23, 2020, at 42.6%, but persisted until mid-February 2021. During the sampling period of this study, an average of 33.4% of South Dakotan participants reported symptoms of either anxiety or depression, peaking on November 11-23, 2020, at 42.3%. The South Dakota data align with national trends, suggesting widespread mental health concerns.

Extensive research has shown the cyclical relationship between stress and alcohol use. Acute alcohol consumption, through modulation of the neurotransmitter γ-aminobutyric acid (GABA), temporarily alleviates symptoms associated with stress and anxiety [26]. Chronic alcohol use, however, increases negative affect, especially during withdrawal, leading to a preoccupation with the seeking of more alcohol [27]. Despite negative consequences, the cycle of alcohol use may continue into alcohol use disorder [28]. The consequences of this are devastating, leading to serious health complications and death [28]. Developing public health surveillance systems that can monitor problematic alcohol use rapidly enough to develop intervention programs before these serious consequences occur is critical for improving population health.

Finally, alcohol-induced mortality increased by 41% from 2019 to 2020 in the state of South Dakota according to the Centers for Disease Control and Prevention (CDC) [29]. According to the State of South Dakota Department of Health, chronic liver disease and cirrhosis, 1 of the leading causes of death attributed to alcohol misuse, increased by 53% from 2019 to 2020 [30]. Unfortunately, deaths due to chronic liver disease and cirrhosis have more than doubled from 2019 to 2021. In 2019, chronic liver disease and cirrhosis were the tenth-leading cause of death in the state; by 2021, they jumped to the seventh-leading cause of death. Although full statistics detailing alcohol-related deaths are not yet available for 2021, alcohol misuse was associated with increased mortality in the state since the beginning of the pandemic. The establishment of wastewater monitoring for drug and alcohol misuse integrated with other metrics, such as helpline call volumes, may be a critical tool for alerting public health officials of the need for SUD treatments, including connecting people with resources, such as call helplines.

### Comparison With Prior Work

The peak in anxiety or depressive symptoms in South Dakotans also corresponds with the peak number of COVID-19 cases during this period. The number of COVID-19 cases reported to the South Dakota Department of Health during the study period peaked on November 12, 2020, at 1960 cases [23]. Of note, peak EtG levels were found approximately 1 week after the peak in COVID-19 cases. Prior research has found that depression and anxiety levels peak at similar times as COVID-19 cases [7]. Finally, others have found that pandemic-associated distress was also associated with problematic alcohol consumption [31,32]. For example, Gilley et al [33] found that alcohol consumption was associated with COVID-19 positivity. These surveys, combined with wastewater analysis, could provide critical information about stress and other potentially harmful behaviors due to the pandemic or other health crises. The information obtained from wastewater analysis and other metrics could help determine spatial and temporal patterns of alcohol use such that targeted campaigns could be created to connect people most in need of mental health and SUD resources.

### Limitations

The stability of alcohol metabolites in wastewater must be considered. Previous studies suggest that EtS is a more stable biomarker of ethanol consumption than EtG [34,35]. This property may play an important role in our findings and must be considered. Given its lack of stability, EtG was more likely to be excreted during the early morning hours, especially on Tuesdays. This may represent a population with a greater risk of developing problematic alcohol consumption behaviors. In contrast, EtS levels may be more inclusive of all alcohol consumers, including social drinkers as well as those with the potential for problematic drinking.

Indeed, this study found that Tuesday levels of EtG but not EtS were significantly correlated with COVID-19 cases and negatively correlated with mental health–related calls to the helpline. No such correlates between COVID-19 cases and alcohol metabolites occurred on Fridays, when social drinking is more common. Continued research is needed to explore these metabolites with indicators of misuse.

Finally, more complex analysis was not conducted in this study. Given the preliminary nature of the research, complex analysis, such as time series with lags incorporated, would be premature. Future research is necessary to develop more complex modeling.

### Conclusion

Although this research was formative, findings from this study suggest that helpline calls during the observed period were associated with housing and health care inquires but not mental health/SUD concerns. However, alcohol consumption metrics were negatively associated with mental health/SUD inquires to the helpline. These findings highlight the critical need for using multiple public surveillance metrics of population health to provide a better understanding of community needs. Tailoring public health announcements around these metrics to direct people to mental health resources would provide a precision medicine approach to promoting positive coping strategies. Finally, although more research is needed, these findings suggest that wastewater surveillance may be helpful in monitoring and impacting population health metrics beyond that of SARS-CoV-2.

## Abbreviations

EtG: ethanol glucuronide
EtS: ethanol sulfate
SUD: substance use disorders
UHPLC-MS-MS: ultrahigh-pressure liquid chromatography coupled with tandem quadrupole mass spectrometry

## Data Availability

The COVID-19 and helpline data analyzed in the study are on the South Dakota Department of Health website and helpline website. Alcohol wastewater data for the study are not publicly available due to community privacy reasons but are available from the corresponding author upon reasonable request.
